# Catechins from oolong tea improve uterine defects by inhibiting STAT3 signaling in polycystic ovary syndrome mice

**DOI:** 10.1186/s13020-020-00405-y

**Published:** 2020-11-30

**Authors:** Ge Hong, Hao Wu, Shi-Tang Ma, Zhe Su

**Affiliations:** 1 Institute of Biomedical Engineering, Chinese Academy of Medical Sciences and Peking Union Medical College, Tianjin Key Laboratory of Biomedical Material, Tianjin, 300192 China; 2 Life and Health College, Anhui Science and Technology University, Fengyang, 233100 China; 3 Department of Emergency, The First Affiliated Hospital of Nanjing Medical University, Nanjing, 200192 China; 4 Tianjin Institute for Drug Control, Tianjin, 300000 China

**Keywords:** Catechins, Uterine defects, Inflammation, STAT3 signaling, Polycystic ovary syndrome

## Abstract

**Background:**

It is showed that inflammation is causative factor for PCOS, leading to a decline in ovarian fertility. Previous studies have reported that tea consumption can reduce the incidence of ovarian cancer. We speculate that catechins from oolong tea (*Camellia sinensis (L.) O.* Kuntze) may have a potential therapeutic effect on PCOS. This study aims to investigate the effects of oolong tea catechins on the uterus of polycystic ovary syndrome (PCOS) mice induced by insulin combined with human chorionic gonadotropin (hCG).

**Methods:**

Sixty female mice were divided into 6 groups (n = 10): model, model + Metformin 200 mg/kg, model + catechins 25 mg/kg, model + catechins 50 mg/kg, and model + catechins 100 mg/kg. Another forty female mice were divided into 4 groups (n = 10): control, control + catechins 100 mg/kg, model, and model + catechins 100 mg/kg. Ovarian and uterine weight coefficients, sex hormone levels, glucose metabolism and insulin resistance, and ovarian and uterine pathology were examined. Changes in NF-κB-mediated inflammation, MMP2 and MMP9 expressions, and STAT3 signaling were evaluated in the uterus of mice.

**Results:**

Catechins could effectively reduce the ovarian and uterine organ coefficients, reduce the levels of E2, FSH and LH in the blood and the ratio of LH/FSH, and improve glucose metabolism and insulin resistance in PCOS mice induced by insulin combined with hCG. In addition, catechins could significantly down-regulated the expression of p-NF-κB p65 in the uterus and the protein expressions of the pro-inflammatory factors (IL-1β, IL-6, and TNF-α). The expressions of mmp2 and mmp9 associated with matrix degradation in uterine tissue were also significantly down-regulated by catechins. Further, catechins significantly reduced the expression of p-STAT3 and increased the expression of p-IRS1 and p-PI3K in the uterus of PCOS mice.

**Conclusion:**

Catechins from oolong tea can alleviate ovarian dysfunction and insulin resistance in PCOS mice by inhibiting uterine inflammation and matrix degradation via inhibiting p-STAT3 signaling.

## Introduction

Polycystic ovary syndrome (PCOS) is a complex reproductive endocrine metabolic syndrome in gynecological clinic with a prevalence of up to 15% in women of child-bearing age [1, 2], and its clinical manifestations are diverse [3], such as rare menstruation or amenorrhea, anovulatory menstrual cycles, sex hormone disorders, insulin resistance, metabolic syndrome, psychological abnormalities, and infertility [4]. Clomiphene is currently the first-line treatment for PCOS-induced infertility [5]. However, clomiphene has the characteristics of high ovulation rate and low pregnancy rate [6], and even has side effects of ovarian hyperstimulation syndrome [7]. Therefore, it is extremely necessary to find a more effective healthcare agent with fewer side effects for the treatment of PCOS [5].

Many studies have reported the healthcare effects of oolong tea and its extracts on dyslipidemia [8], obesity [9] and insulin resistance [10]. Catechins and other major components of oolong tea have been shown to have various healthcare effects such as antioxidant stress and anti-inflammatory [11, 12]. At present, many studies have proved that insulin resistance and abnormal glucose metabolism are the major pathogenesis of PCOS [13]. Insulin resistance in the ovaries of PCOS patients can cause ovarian dysfunction and endocrine abnormalities [14–16]. Investigations have also shown that inflammation has become another causative factor [17, 18], which can affect the growth and development of follicles in the ovaries of patients with PCOS [19], leading to a decline in ovarian fertility [20]. Therefore, we speculate that the extracts of catechins from oolong teas may have a potential therapeutic effect on PCOS. Previous studies have reported that tea consumption can reduce the incidence of ovarian cancer [21, 22], which may suggest tea extracts may be a promising candidate for preventing PCOS.

An increasing number of clinical studies indicate that impaired endometrial function leads to recurrent miscarriages, preterm births, endometrial hyperplasia, and canceration in patients with PCOS [23]. Other studies have shown an association between many endometrial cell lines and inflammation-related molecular disorders under PCOS conditions [24]. The aim of this study was to investigate the effects of catechins on PCOS mice induced by sex hormone disorders and insulin resistance. In the mouse model we used a combination of insulin and human chorionic gonadotropin (hCG) to induce PCOS-like features [25]. In this model, we focus on the effects of catechins extracts from oolong tea on PCOS-like symptoms, glucose metabolism, insulin resistance, and pathological changes in the ovaries, as well as on the changes of uterine inflammation-related molecules, endometrial matrix degradation, and uterine STAT3 signaling in mice. Our results confirm the significant healthcare effect of catechins on uterine dysfunction in PCOS mice.

## Materials and methods

### Catechins extract prepared

**Fig. 1:**
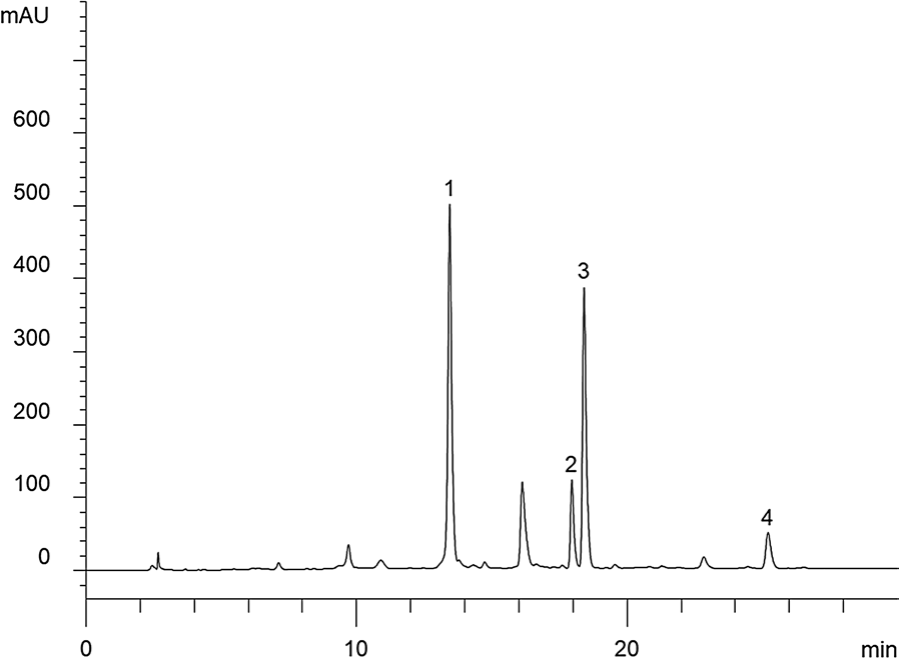
Representative HPLC chromatograms of the extracts (catechins) of catechins. 1. Epigallocatechin (22.4 mg/g), 2. Epicatechin (5.9 mg/g), 3. Catechin (17.68 mg/g), 4. Epicatechin gallate (3.86 mg/g)

Epigallocatechin (GD738X06), epicatechin (E68206), catechin (E68206V) and epicatechin gallate (C315884S) were purchased from Anhui cool Bioengineering Co., Ltd (Anhui, China). The Tieguanyin oolong tea-leaves were collected in Anxi County, Fujian Province, China. The catechins in oolong tea-leaves were extracted as follows: Firstly, oolong tea-leaves were washed with distilled water, dried at 37 ℃, and then lyophilized. The dried oolong tea-leaves were ground into a powder using a blender. Lyophilized powder (10 g) was mixed in 100 ml of 75/25 water/methanol. The mixture was incubated at 80 ℃ for 2 h. After filtering the solids, the filtered liquid was concentrated under vacuum to obtain the extracts. According to the total amount of catechins, the quality of the extracts can be quality controlled (see Figs. 1, 4 kinds of catechins were detected from the extracts, the content of 4 kinds of catechins was 49.84 mg/g extracts), and the total extracts was used for gavage at a dose of 100 mg/kg in mice, the dose of catechins (including epigallocatechin, epicatechin, catechin and epicatechin gallate) was about 4.984 mg/kg.

The detection methods for the four substances are as follows: the four main ingredients of extracts of catechins were determined using an Agilent 1260 liquid chromatography system [26]. Briefly, 10 μL extracts was injected into the apparatus with an auto sampler. Chromatographic separation was achieved at a flow rate of 1 mL/min with an Agilent Zorbax SB-C18 column (4.6 × 250 mm, 5 μm). The mobile phase was composed of solvent A (0.1% phosphoric acid) and solvent B (acetonitrile). The linear gradient solution was performed from 2 to 8% solvent B for 0–7 min, 8–15% solvent B for 7–15 min, 15–19% solvent B for 15–27 min, 19–40% solvent B for 27–30 min. The separation temperature was 40 ℃, with a detection wave length of 210 nm. A representative HPLC chromatogram of catechins extracted from oolong tea was shown in Fig. 1.

### Reagents

Anti-NF-kB p65 antibody (ab16502), Anti-NF-kB p65 (phospho S536) antibody (ab86299), Anti-IL-1 beta antibody (ab9722), Anti-IL-6 antibody (ab7737), Anti-TNF Alpha antibody (ab1793), Anti-MMP2 antibody (ab97779), Anti-MMP9 antibody (ab38898), Anti-STAT3 antibody 9D8 (ab119352), Anti-STAT3 (phospho Y705) antibody (ab76315), Anti-IRS1 antibody (ab131487), Anti-IRS1 (phospho S307) antibody (ab1194), Anti-PI 3 Kinase p85 alpha antibody (ab86714), Anti-PI 3 Kinase p85 alpha (phospho Y607) antibody (ab182651), and Anti-GAPDH antibody(ab181602) were purchased from abcam (Cambridge, UK). HRP-conjugated Affinipure Goat Anti-Rat IgG(H + L) (SA00001-15) was purchased from proteintech (Wuhan, China). Hematoxylin–Eosin/HE staining kit (G1120) and DAB color development kit (DA1010) were purchased from Solarbiolife sciences (Beijing, China). Mouse LH (Luteinizing Hormone) ELISA kit(E-EL-M0057c), Mouse FSH (Follicle Stimulating Hormone) ELISA kit (E-EL-M0511c), Mouse E_2_ (17-βestradiol) ELISA kit (E-EL-0150c), and Mouse insulin ELISA kit (E-EL-M1382c) were purchased from Elabscience (Wuhan, China).

### Experimental animals

Eight-week-old female C57BL/6 mice were purchased from Shanghai Slark Experimental Animal Co., Ltd (Shanghai, China). All experimental procedures and experimental animal licenses were approved by the Experimental Animal Ethics Committee of Anhui Science and Technology University. Animals were housed in groups, with free access to food and water. The laboratory temperature was 22 ± 2 ℃ with a 12 h light/dark cycle.

Sixty female mice were randomly divided into normal mice group (control), insulin and human chorionic gonadotropin treated mice group (model), 200 mg/kg metformin treated model mice group, 25 mg/kg catechins treated model mice group, 50 mg/kg catechins treated model mice group, and 100 mg/kg catechins treated model mice group. There were 10 mice in each group. Another forty female mice were randomly divided into normal mice group (control, n = 10), 100 mg/kg catechins treated normal mice group (control + catechins, n = 10), insulin and human chorionic gonadotropin treated mice group (model, n = 10), and 100 mg/kg catechins treated model mice group (model + catechins, n = 10).

Insulin subcutaneous injections started at 0.5 IU/day, increased by 0.5 IU per day, and stopped increasing doses to 6.0 IU/day to induce hyperinsulinemia and insulin resistance in mice. At the same time, 6.0 IU/day human chorionic gonadotropin (hCG) was injected subcutaneously twice a day to induce hyperandrogenemia in mice. After 21 days, model mice were randomly divided into model and model + catechins groups. Extracts of catechins were administered orally to normal mice and model mice at 100 mg/kg once a day for 1 month.

### Body weight and uterine and ovarian organ coefficient

At the end of the treatment cycle, the weight of the mice in each group was measured. After the mice were anesthetized, the uterus and ovaries of the mice were taken, and the weight of the bilateral ovaries and the weight of the Y-shaped uterus of the mice were weighed using a 1/10,000 analytical balance (Mettler toledo, ME204). Then the uterine and ovarian organ coefficient was calculated as follows: uterine organ coefficient = (uteri weight/body weight) × 100; ovarian organ coefficient = (ovarian weight/body weight) × 100.

### Determination of hormone levels

At the end of the treatment cycle, and after fasting, the blood of the mice was collected. With reference to manufacturer's manual, the levels of LH, FSH, E_2_, and insulin in the serum of each group of mice were detected by using enzyme-linked immunosorbent assay (ELISA) kit.

### Blood glucose, oral glucose tolerance test (OGTT) and insulin resistance

The fasting blood glucose of the mice was also measured using a glucose content detection kit (trace method) (Solarbio life sciences, BC2505). The oral glucose tolerance test was performed on the mice in each group, and the blood glucose level of the mice was measured after glucose was given after 0, 30, 60, 90, 120 min. According to the following formula, Homeostatic model assessment of insulin resistance (HOMA-IR) = fasting blood glucose × fasting serum insulin/22.5.

### Histology and immunostaining

The ovaries and uterine tissues from above experimental animals were fixed in 10% formalin (Sinopharm Chemical Reagent Co., Ltd,10,010,061), embedded in paraffin (Sinopharm Chemical Reagent Co., Ltd, 69,018,961), and the mouse ovary and uterus were cut into 5 µm using a paraffin microtome (Leica Microsystems, RM2235). The sections were stained according to the HE staining procedure. After sealing with a neutral resin (Solarbiolife sciences, G8590), the slices were photographed using an optical microscope (Leica Microsystems, DMILLED) [27].

Immunohistochemical procedures have been performed on sections of mouse uterus for dewaxing, rehydration, antigen retrieval, and endogenous catalase extinguishing. Sections were incubated with primary antibody (Anti-NF-kB p65 (phospho S536) antibody (ab86299), Anti-MMP2 antibody (ab97779), Anti-MMP9 antibody (ab38898), recombinant Anti-STAT3 (phospho Y705) antibody (ab76315)) in an immunohistochemical wet box at 4 °C overnight. HRP-modified secondary antibody was then used to incubate in an immunohistochemical wet box at 4 °C for two hours. Then the sections were treated with DAB color development kit for 10 min. After washing the sections with TBST, hematoxylin was used to stain the nuclei of uterine sections. Then neutral resin was used to seal the sheet. The sections were photographed using an optical microscope (Leica Microsystems, DMILLED).

### Western blot analysis

After anesthetizing the mice in each group, the mouse uterus was taken, and the total protein was extracted according to the instructions using enhanced BCA protein assay kit (Beyotime, P0010), and the protein concentration was determined. Referring to the detailed description of the western blot protocol [28], an equal amount (50 μg) of protein isolated from mouse uterine tissue was separated on an SDS-PAGE gel, and then the protein was transferred to a PVDF membrane. The primary antibody is diluted according to the instructions, including Anti-NF-kB p65 antibody, Anti-NF-kB p65 (phospho S536) antibody, Anti-IL-1 beta antibody, Anti-IL-6 antibody, Anti-TNF alpha antibody, Anti-MMP2 antibody, Anti-MMP9 antibody, Anti-STAT3 antibody, recombinant Anti -STAT3 (phospho Y705) antibody, Anti-IRS1 antibody, Anti-IRS1 (phospho S307) antibody, Anti-PI 3 Kinase p85 alpha antibody, Anti -PI3Kinase p85 alpha (phospho Y607) antibody, and GAPDH. The PVDF membrane was incubated with the primary antibody overnight, washed three times with TBST, and then incubated with the HRP-conjugated secondary antibody for two hours, and washed three times with TBST again. A gel imaging system (Bio-Rad,GelDoc XR +) was used to obtain a blot of each protein.

### Statistical analysis

For the data detected by the kit, a standard curve was drawn and the detection values of each index of the sample were calculated. For immunohistochemically stained pictures, the average optical density values of each picture were analyzed using image pro plus 6.0. For western blot images, the gray value of each protein was analyzed using image lab. For all measurement data obtained, the data were expressed as mean ± SD. Statistical analysis was performed on all data using SPSS 22.0 software (SPSS Inc., Chicago, IL). Shapiro–Wilk test was used to determine whether the data were normally distributed. One-way analysis of variance was used to analyze the differences between groups. Tukey's post hoc test was performed on the normal distribution data. Graph pad 7.0 is used for mapping and calculating AUC for OGTT data. All p < 0.05 were considered statistically significant.

## Results

### Metformin or Catechins treatment reduces uterine weight, hormone secretion and insulin resistance in mice

**Fig. 2:**
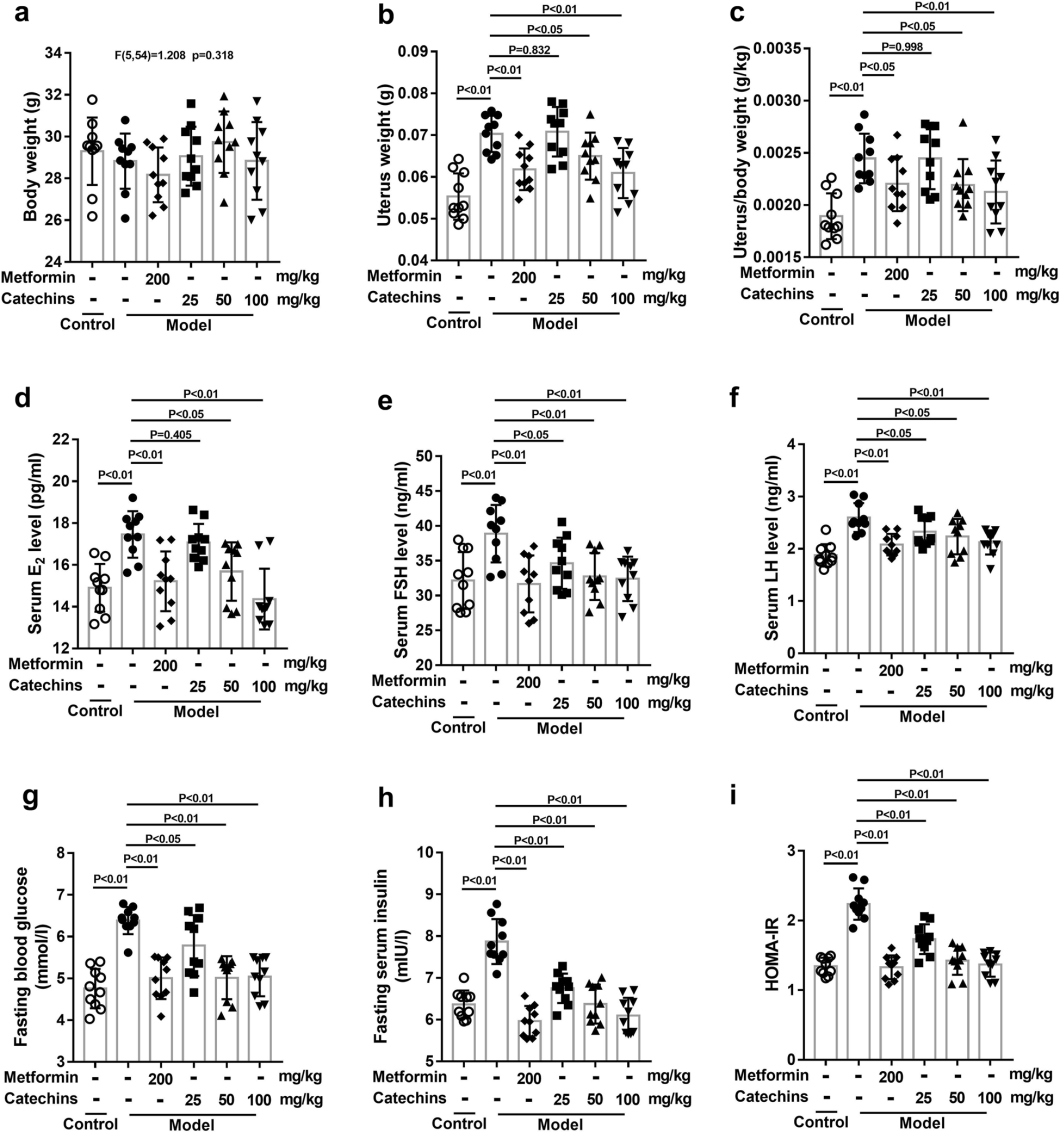
Metformin or Catechins treatment reduces uterine weight, hormone secretion and insulin resistance in mice. **a** Body weight was measured in different groups in mice after 28 days treatment. **b** Uterus weight was measured in different groups in mice after 28 days treatment. **c** Uterus/body weight was calculated in different groups in mice after 28 days treatment. **d** The estrogen content in serum was measured in different groups in mice after 28 days treatment. **e** The follicle stimulating hormone content in serum was measured in different groups in mice after 28 days treatment. **f** The luteinizing hormone content in serum was measured in different groups in mice after 28 days treatment. **g** The fasting blood glucose level was measured in different groups in mice after 28 days treatment. **h** The fasting insulin level in serum was measured in different groups in mice after 28 days treatment. **i** HOMA-IR (Homeostatic model assessment of insulin resistance) was calculated in different groups in mice. HOMA-IR = fasting blood glucose × fasting serum insulin/22.5. N = 10 in each group. A P value of < 0.05 was considered statistically significant

The effect of different concentration of catechins (25, 50, and 100 mg/kg) on the body weight, uterus weight, hormone secretion and insulin resistance were firstly examined in mice, 200 mg/kg metformin was used as a positive drug. Result showed that there were no significant changes in body weight to each group (Fig. 2a). The mice in model group treated with insulin and hCG showed an increase in uterus weight and in organ coefficients of uterus. Metformin and different concentration of catechins treatment significantly reduced uterus weight, and organ coefficients of uterus in model mice (Fig. 2b–c). We then collected blood from mice in each group and tested the levels of E2, FSH, and LH. The mice in model group showed increased levels of E2, FSH, LH. After metformin and different concentration of catechins treatment, the levels of E2, FSH, and LH were significantly reduced (Fig. 2d–f). We observed that fasting blood glucose, fasting insulin and were significantly increased in the mice of model group compared to the mice of control group, and metformin and different concentration of catechins reduced them in model mice (Fig. 2g–i). The above results show that like metformin, catechins reduces uterine weight, hormone secretion and insulin resistance in insulin and human chorionic gonadotropin treated mice.

### Catechins improves PCOS-like symptoms and insulin resistance in model mice

**Fig. 3:**
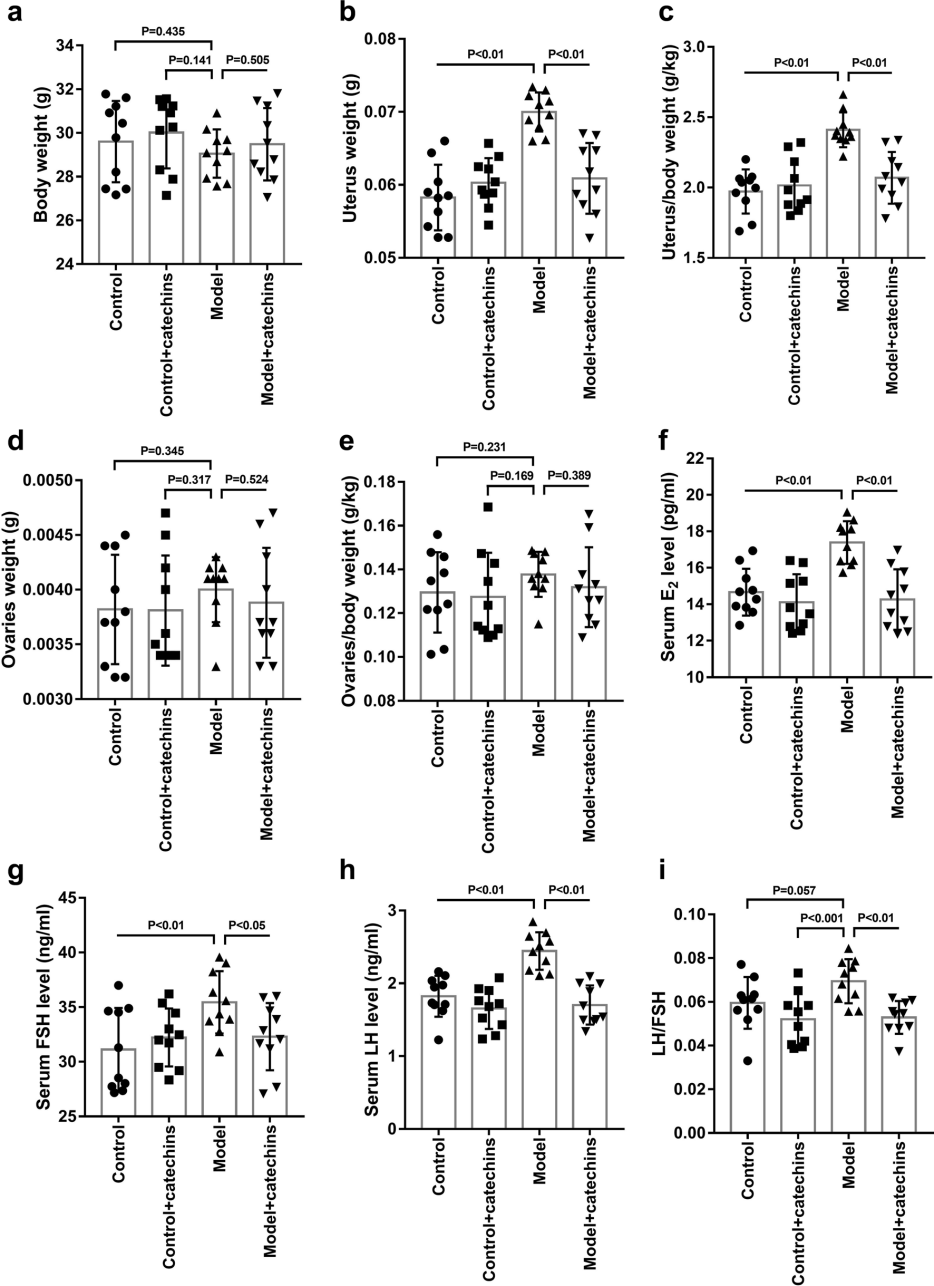
Catechins improves PCOS-like symptoms in model mice. **a** Body weight was measured in control or model mice with or without 100 mg/kg catechins treatment for 28 days. **b** Uterus weight was measured in control or model mice with or without 100 mg/kg catechins treatment for 28 days. **c** Uterus/body weight was calculated in different groups in mice. **d** Ovaries was measured in control or model mice with or without 100 mg/kg catechins treatment for 28 days. **e** Ovaries /body weight was calculated in different groups in mice. **f** The estrogen content in serum was measured in control or model mice with or without 100 mg/kg catechins treatment for 28 days. **g** The follicle stimulating hormone content in serum was measured in control or model mice with or without 100 mg/kg catechins treatment for 28 days. **h** The luteinizing hormone content in serum was measured in control or model mice with or without 100 mg/kg catechins treatment for 28 days. **i** Luteinizing hormone/follicle stimulating hormone was calculated in different groups in mice. N = 10 in each group. A P value of < 0.05 was considered statistically significant

Then the effect of catechins (100 mg/kg, oral) on the body weight, uterus weight, and ovaries weight in mice was examined in another separate experiment. It is showed that there were no significant changes in body weight to each group (Fig. 3a). The mice in model group treated with insulin and hCG showed an increase in uterus weight and ovaries weight, and an increase in organ coefficients of uterus and ovaries. After catechins treatment in model mice, uterus weight, ovaries weight, and organ coefficients of uterus and ovaries were significantly reduced, whereas these changes were not seen in normal mice given catechins (Fig. 3b–e). We then collected blood from mice in each group and tested the levels of E2, FSH, and LH. The mice in model group showed increased levels of E2, FSH, LH. And LH/FSH values also significantly increased in the mice of model group, which indicated that LH increased more significantly in these model mice. After catechins treatment, the levels of E2, FSH, LH and LH/FSH were significantly reduced. The levels of these detection factors were close to those of normal mice in the control group or control + catechins group (Fig. 3f–i). The above results show that insulin combined with hCG causes uterine and ovarian enlargement in mice, as well as fluctuations in sex hormones, and catechins can reduce this situation in model mice. In addition, catechins have no effect on body weight, uterus and ovaries weight, and levels of sex hormone of normal mice.

**Fig. 4:**
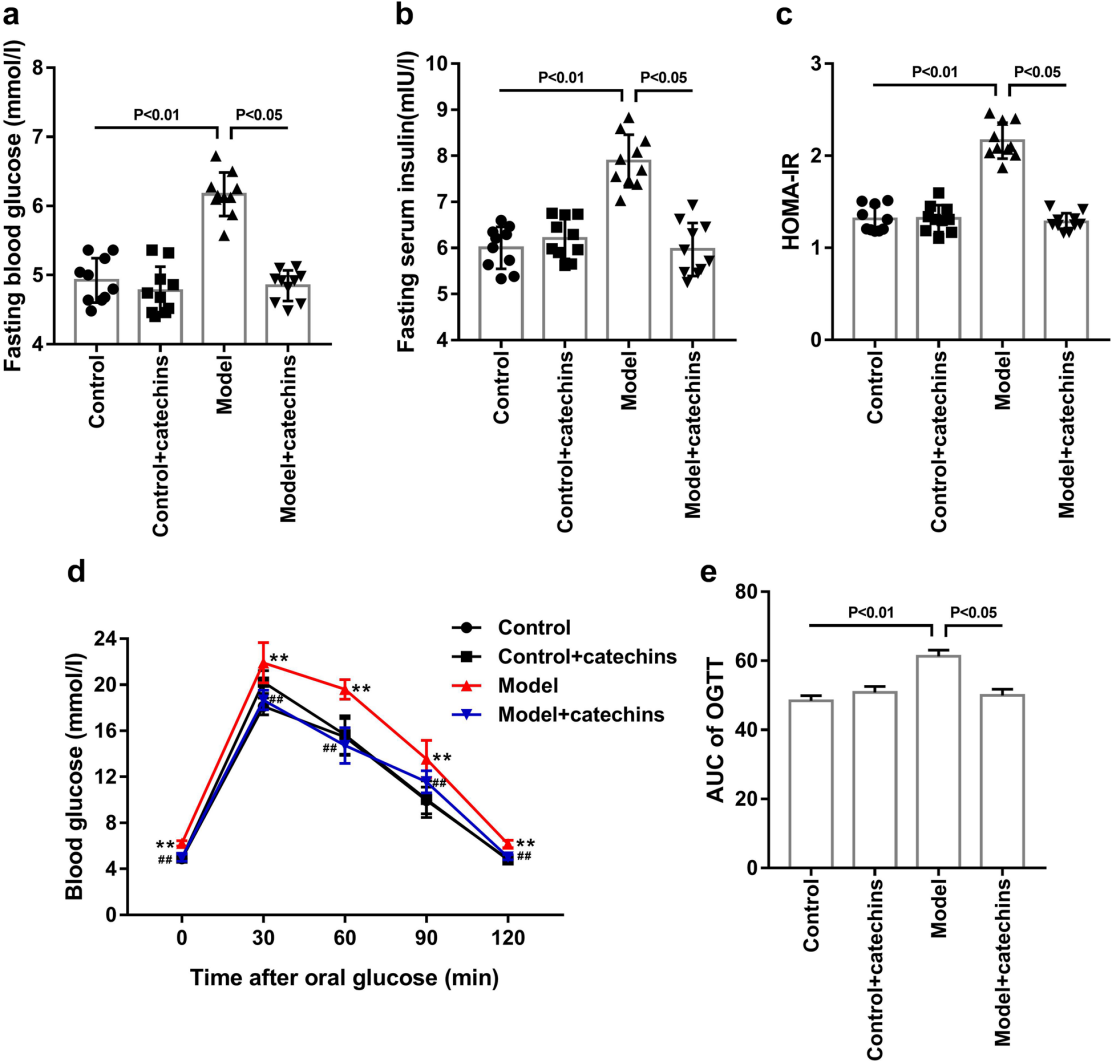
Catechins improves insulin resistance in model mice. **a** The fasting blood glucose level was measured in control or model mice with or without 100 mg/kg catechins treatment for 28 days. **b** The fasting insulin level in serum was measured in control or model mice with or without 100 mg/kg catechins treatment for 28 days. **c** HOMA-IR (Homeostatic model assessment of insulin resistance) was calculated in different groups in mice. HOMA-IR = fasting blood glucose × fasting serum insulin/22.5. **d** The OGTT (oral glucose tolerance test) was performed in control or model mice with or without 100 mg/kg catechins treatment for 28 days. The blood glucose level was measured after glucose was given after 0, 30, 60, 90, 120 min in different groups in mice. **e** The AUC (area under the curve) was calculated in different groups in mice. N = 10 in each group. A P value of < 0.05 was considered statistically significant

We observed that fasting blood glucose and fasting insulin were significantly increased in the mice of model group compared to the mice of control group and control + catechins group (Fig. 4a–b). We calculated the insulin resistance coefficient and the results showed that mice in model group had a significantly higher insulin resistance coefficient (Fig. 4c). The results of the OGTT experiment showed a decrease in glucose tolerance of the model mice (Fig. 4d), which was shown by an increase in AUC of OGTT (Fig. 4e). Moreover, compared with the model group, the fasting blood glucose, fasting insulin, HOMA-IR, and glucose tolerance in the model + catechins group were significantly reduced (Fig. 4). The above results show that catechins improves PCOS symptoms and insulin resistance in model mice.

### Catechins improves ovarian and uterine morphology in model mice

**Fig. 5:**
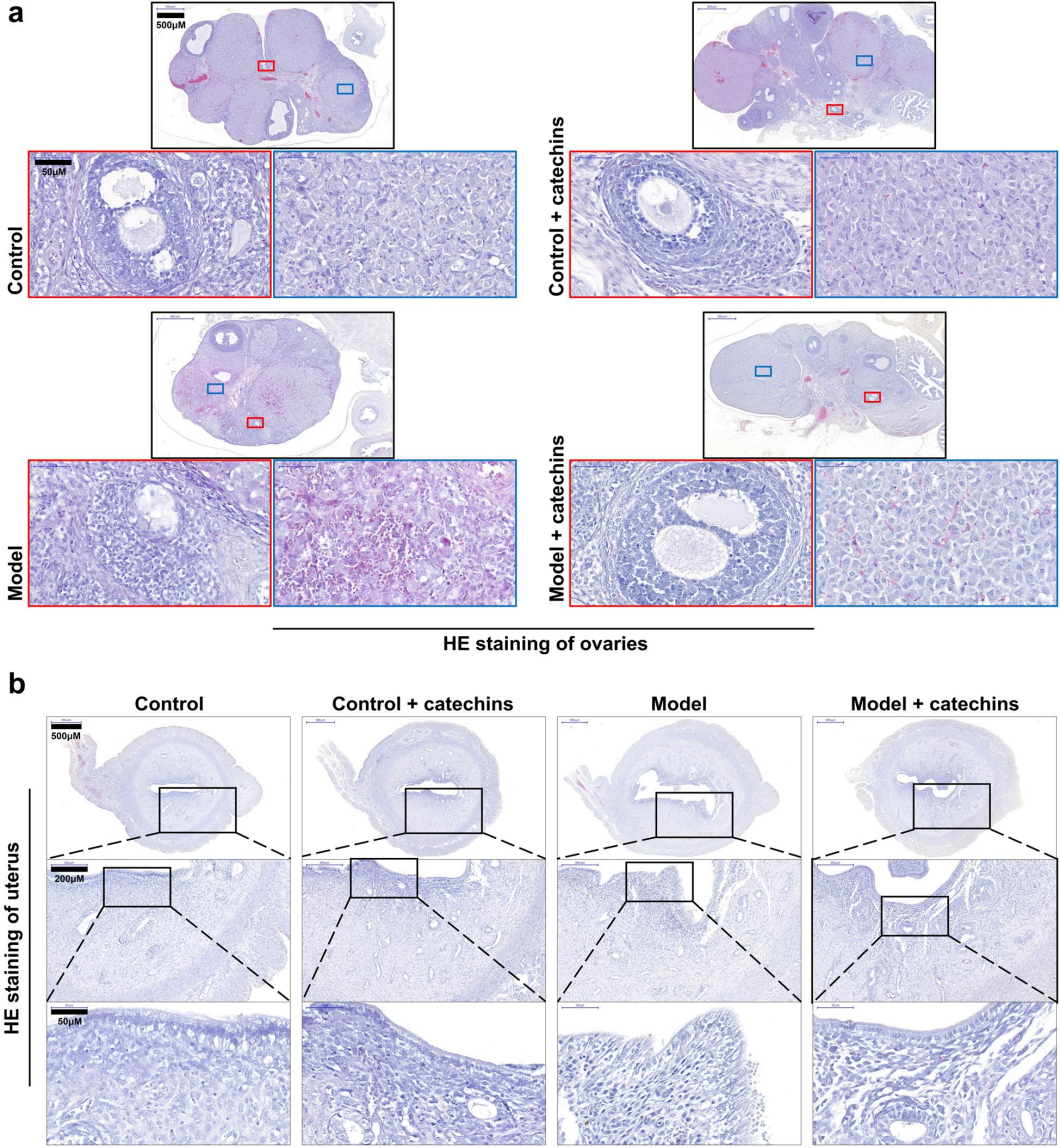
Catechins improves pathological damage of ovaries and uterus in model mice. **a** The HE (hematoxylin and eosin) staining of ovaries was performed in control or model mice with or without 100 mg/kg catechins treatment for 28 days. Different magnifications of representative images were displayed. Scale bar in low magnification = 500 μm. The boxed area in red and blue color were shown by a higher magnification. Scale bar in high magnification = 50 μm. **b** The HE staining of uterus was performed in control or model mice with or without 100 mg/kg catechins treatment for 28 days. Different magnifications of representative images were displayed. Scale bar in low magnification = 500 μm. The boxed area in black color were shown by a higher magnification. Scale bar in higher magnification = 200 μm. Scale bar in highest magnification = 50 μm. N = 6 in each group

The ovarian follicle structure of mice in control group and control + catechins group was clear, and the corpus luteum and differently developed follicles (Fig. 5a, low magnification) can be seen. The follicular wall was full of densely packed and multi-layered granular cells, the follicular membrane was spindle-shaped (Fig. 5a, high magnification, left panel), and membrane-interstitial was non-proliferative (Fig. 5a, high magnification, right panel) in control group and control + catechins group. The ovaries of mice in the model group showed cystic sinus follicles, enlarged sinus cavity, reduced granular cell layer and loose arrangement, and oocytes disappearance (Fig. 5a, high magnification, left panel), decreased corpus luteum tissue, with luteinization, significantly cell proliferation(Fig. 5a, high magnification, right panel).In the model + catechins group, a small amount of cystic sinus follicles was found in the ovarian tissue of the mice, the sinus cavity was small, and the number of corpus luteum was less than that in the control group. However, compared with the mice in model group, the granulosa cell layer of the ovary is thicker, complete, and tightly arranged (Fig. 5a).

The histological analysis of the uterus showed that the uterine cavity epithelial cells of mice in the control group and the control + catechins group remained cubic, and the inner wall cells of the uterus were tightly arranged (Fig. 5b, high magnification), the outer diameter of the uterus was smaller (Fig. 5b, low magnification) compared to that of the model group. The uterine diameter of the mice in the model group was larger, which indicates that there was extensive inflammation in the mouse reproductive organs. At the same time, the number of epithelial cells and cell layers in the uterine cavity of the model group were reduced (Fig. 5b, high magnification). The outer diameter of the uterus of the mice in the model + catechins group was similar to that of the control group and the control + catechins group, and was slightly smaller than that of the model group. More obvious was that the inner wall of the uterus was densely packed, and the number of luminal epithelial cells and cell layers were significantly increased. These results suggested that catechins have obvious healthcare effects on the ovaries and uterus of PCOS mice induced by insulin combined with hCG, and the therapeutic effect of catechins on PCOS may depend on its inhibitory effect on inflammation of the reproductive organs.

### Catechins inhibits NF-κB-mediated inflammation in the uterus of model mice

**Fig. 6:**
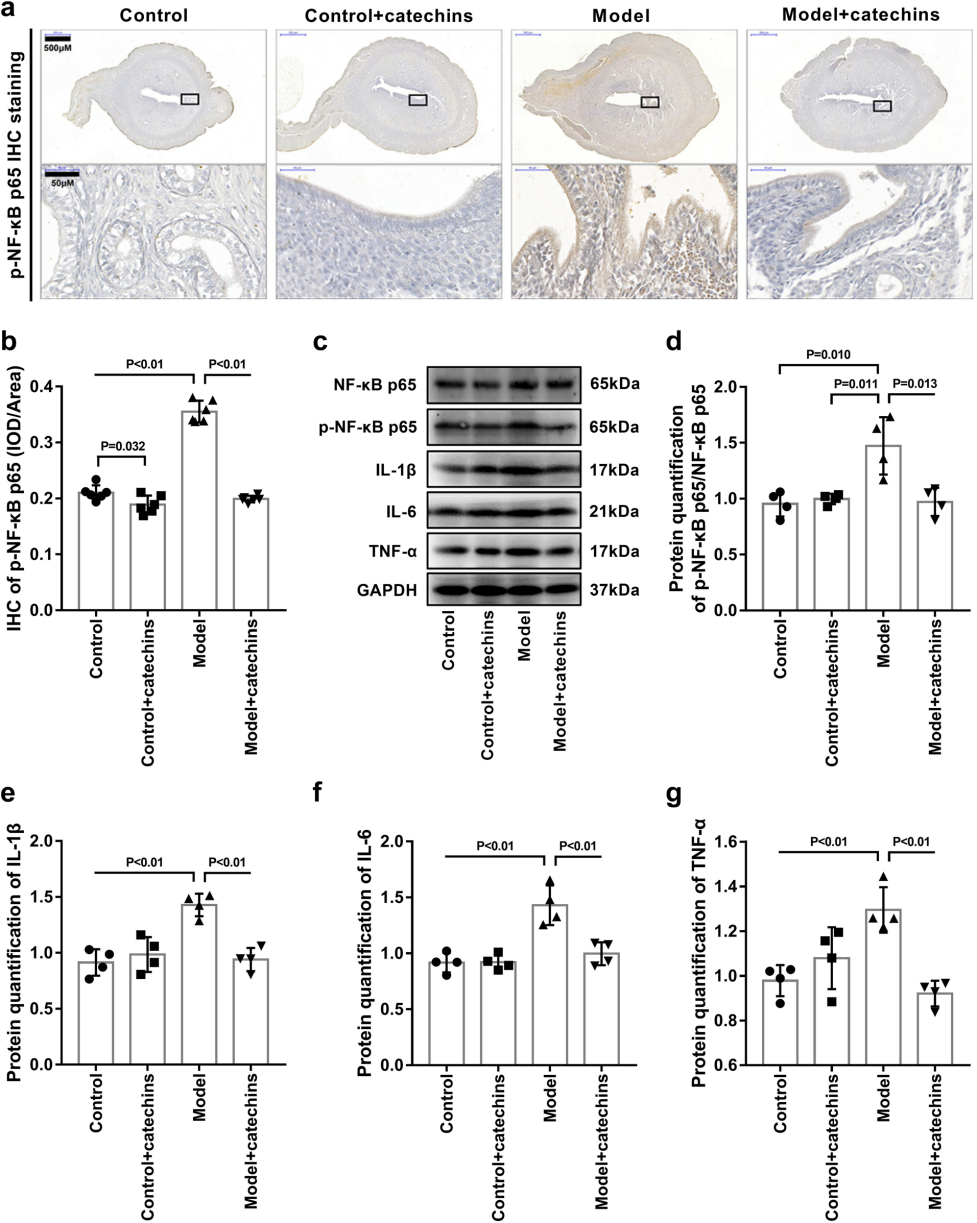
Catechins inhibits NF-κB-mediated inflammation in the uterus of model mice. **a** The immunohistochemical staining of p-NF-κB p65 was performed in uterus in control or model mice with or without 100 mg/kg catechins treatment for 28 days. Different magnifications of representative images were displayed. Scale bar in low magnification = 500 μm. The boxed area in black color were shown by a higher magnification. Scale bar in high magnification = 50 μm. **b** Related to 5A, the mean optical density analysis of p-NF-κB p65 in uterus was performed in different groups in mice. N = 6 in each group. **c** Representative images of western blot analysis of NF-κB p65, p-NF-κB p65, IL-1β, IL-6 and TNF-α were displayed in uterus in control or model mice with or without 100 mg/kg catechins treatment for 28 days. **d** Related to 5C, the relative quantitative analysis was performed of p-NF-κB p65/NF-κB p65of western blot analysis in uterus in different groups in mice. N = 4 in each group. **e** Related to 5C, the relative quantitative analysis was performed of IL-1β of western blot analysis in uterus in different groups in mice. N = 4 in each group. **f** Related to 5C, the relative quantitative analysis was performed of IL-6of western blot analysis in uterus in different groups in mice. N = 4 in each group. **g** Related to 5C, the relative quantitative analysis was performed of TNF-α of western blot analysis in uterus in different groups in mice. N = 4 in each group. A P value of < 0.05 was considered statistically significant

Further, we performed immunohistochemical and western blot analysis on the uterus of each group of mice to determine the expression level of NF-κB in the uterus. The results showed that compared with the control group and control + catechins group, the expression of p-NF-κB p65 in the uterus of the model group was significantly increased. The expression of p-NF-κB p65 was significantly reduced in model + catechins group (Fig. 6a–d). Moreover, the protein expression of IL-1β, IL-6 and TNF-α in the uterus of model group was significantly increased, which was consistent with the change trend of p-NF-κB p65 expression. catechins treatment reduced IL-1β, IL -6 and TNF-α expression in model mouse uterus (Fig. 6c–g). These results indicated that catechins treatment inhibits the increase of NF-κB-mediated inflammation in the uterus of mice with PCOS.

### Catechins inhibits MMP2 and MMP9 expressions in uterus of model mice

**Fig. 7:**
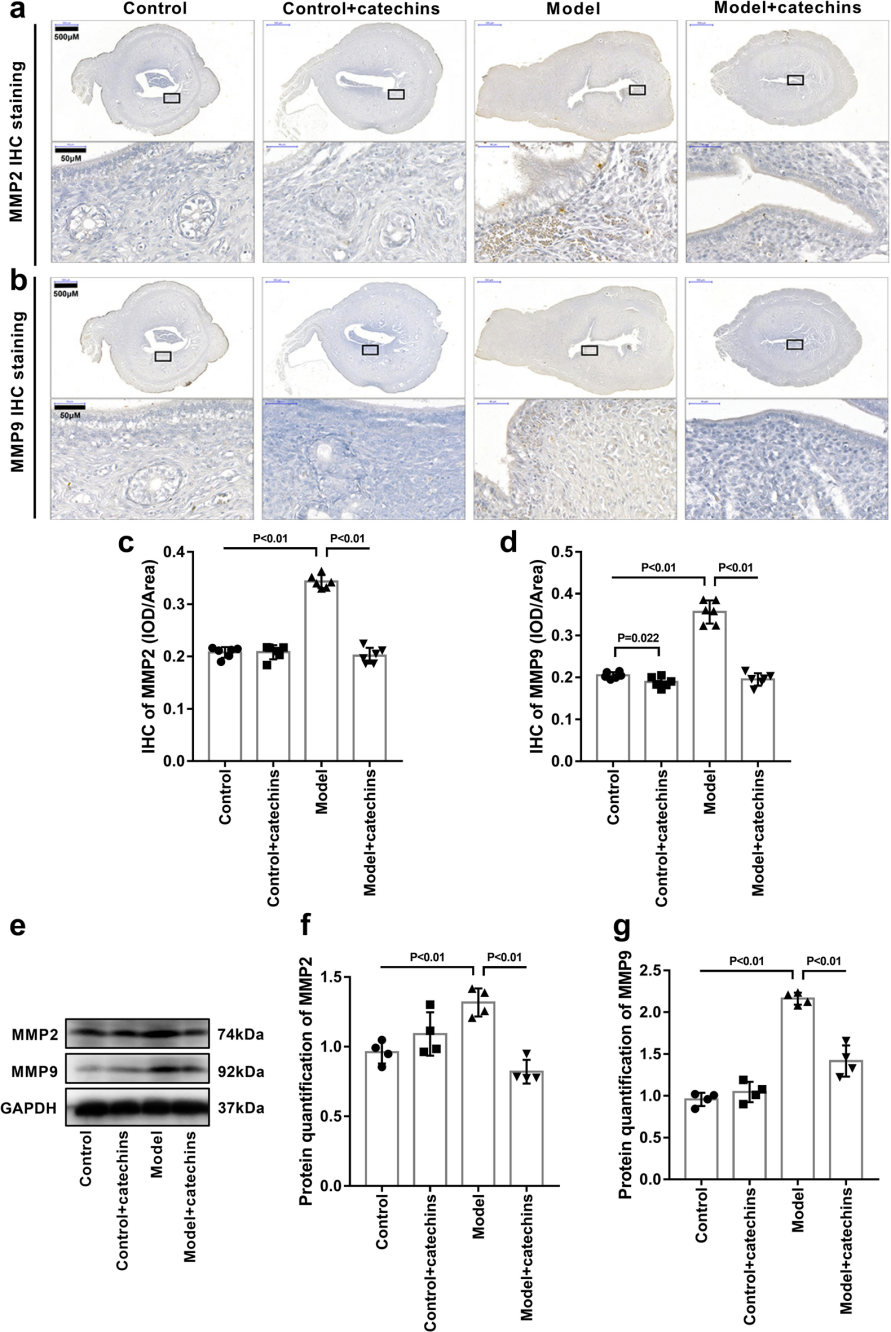
Catechins inhibits MMP2 and MMP9 expressions in uterus of model mice. **a** The immunohistochemical staining of MMP2 was performed in uterus in control or model mice with or without 100 mg/kg catechins treatment for 28 days. **b** The immunohistochemical staining of MMP9 was performed in uterus in control or model mice with or without 100 mg/kg catechins treatment for 28 days. Different magnifications of representative images were displayed. Scale bar in low magnification = 500 μm. The boxed area in black color were shown by a higher magnification. Scale bar in high magnification = 50 μm. **c** Related to 6A, the mean optical density analysis of MMP2 in uterus was performed in different groups in mice. N = 6 in each group. **d** Related to 6B, the mean optical density analysis of MMP9 in uterus was performed in different groups in mice. N = 6 in each group. **e** Representative images of western blot analysis of MMP2 and MMP9 were displayed in uterus in control or model mice with or without 100 mg/kg catechins treatment for 28 days. **f** Related to 6E, the relative quantitative analysis was performed of MMP2of western blot analysis in uterus in different groups in mice. N = 4 in each group. **g** Related to 6E, the relative quantitative analysis was performed of MMP9 of western blot analysis in uterus in different groups in mice. N = 4 in each group. A P value of <0.05 was considered statistically significant

Given that we found a decrease in cell compactness and cell number in the uterine lining of model mice, this may be the result of cell matrix degradation. Therefore, we performed serial sections of the uterus of each group of mice, and performed immunohistochemical staining of MMP2 and MMP9 on these serial sections. The results showed that compared with the control group and control + catechins group, the expressions of MMP2 and MMP9 in the uterus of the model group were significantly increased. And the expressions of MMP2 and MMP9 in the uterus of the catechins treated model mice were significantly reduced (Fig. 7a–c). It should be mentioned here that the expression of MMP9 also showed a decreasing trend after catechins administration in normal mice. Western blots were performed to reconfirm the levels of MMP2 and MMP9 in the uterus. The results of western blot also showed that the expressions of MMP2 and MMP9 proteins in the uterus of model mice were significantly increased, consistent with their changes in immunohistochemical staining. catechins treatment reduced the expression of MMP2 and MMP9 in the uterus of model mice (Fig. 7d–g). These results demonstrated that catechins treatment inhibited MMP2 and MMP9-mediated endometrial damage in the uterus of mice with PCOS.

### Catechins inhibits STAT3 signaling in uterus of mice

**Fig. 8:**
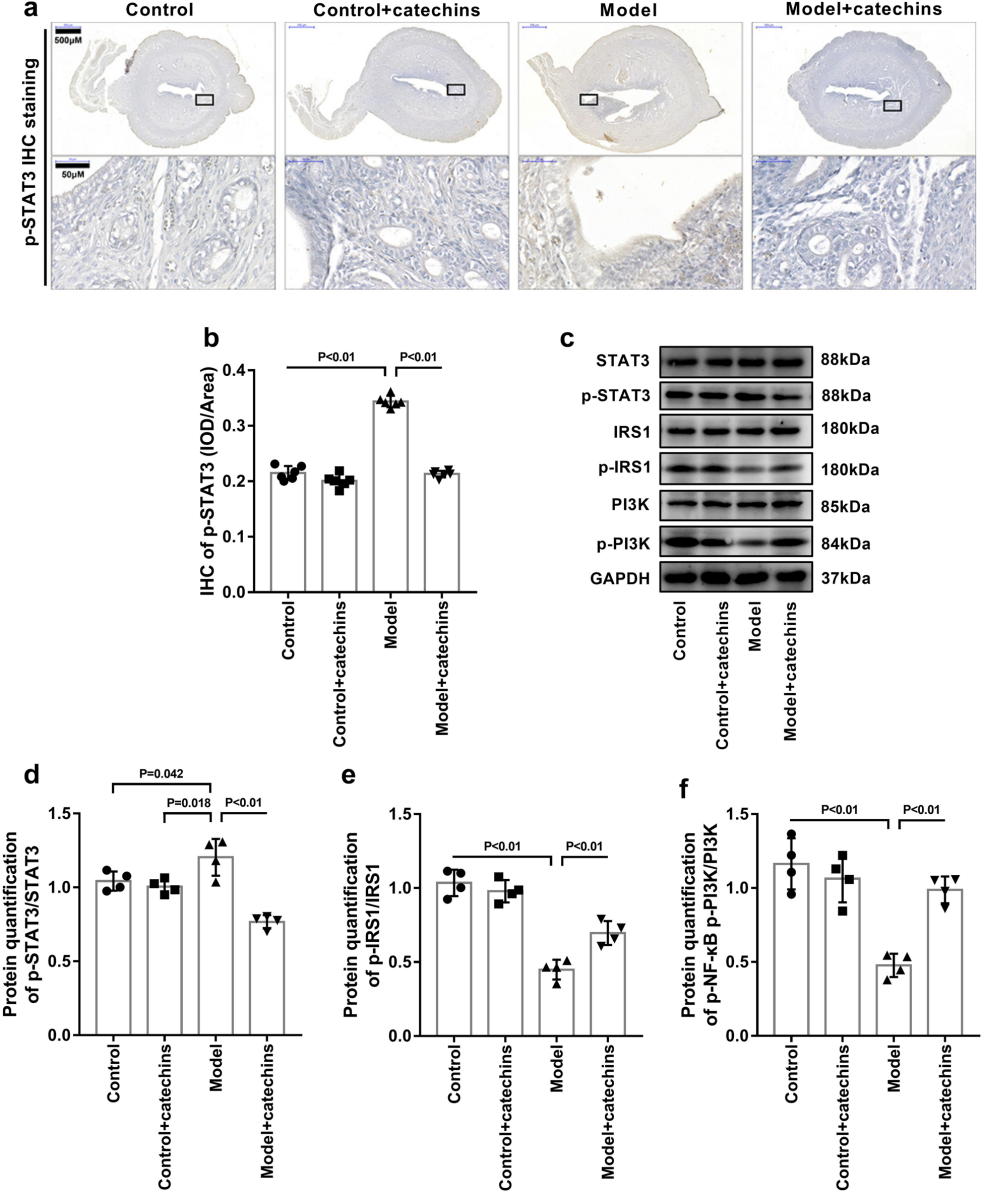
Catechins inhibits STAT3 signaling in uterus of mice. **a** The immunohistochemical staining of p-STAT3 was performed in uterus in control or model mice with or without 100 mg/kg catechins treatment for 28 days. Different magnifications of representative images were displayed. Scale bar in low magnification = 500 μm. The boxed area in black color were shown by a higher magnification. Scale bar in high magnification = 50 μm. **b** Related to 8A, the mean optical density analysis of p-STAT3 in uterus was performed in different groups in mice. N = 6 in each group. **c** Representative images of western blot analysis of STAT3, p-STAT3, IRS1, p-IRS1, PI3K and p-PI3K were displayed in uterus in control or model mice with or without 100 mg/kg catechins treatment for 28 days. **d** Related to 8C, the relative quantitative analysis was performed of p- STAT3/STAT3of western blot analysis in uterus in different groups in mice. N = 4 in each group. **e** Related to 8C, the relative quantitative analysis was performed of p- IRS1/IRS1of western blot analysis in uterus in different groups in mice. N = 4 in each group. **f** Related to 8C, the relative quantitative analysis was performed of p-PI3K/PI3Kof western blot analysis in uterus in different groups in mice. N = 4 in each group. A P value of < 0.05 was considered statistically significant

Next, we investigated the possible mechanisms of catechins-induced inhibition of intrauterine inflammation and matrix degradation in mice, and performed immunochemical staining and western blot analysis to measure the possible signaling that regulated the NF-κB and MMPs signaling systems. We first performed p-STAT3 immunohistochemical staining on the uterus of each group of mice. The results showed that compared with the uterus of mice in control group and control + catechins group, the expression of p-STAT3 in the uterus of the model group was significantly increased. And the expression of p-STAT3 in the uterus of the catechins-treated model mice was significantly reduced. (Fig. 8a–b). At the same time, western blot results confirmed that the expression of p-STAT3 in the uterus was indeed the same as that detected by immunohistochemical staining (Fig. 8c–d). We applied western blot to further detect the changes of IRS1 and PI3K in the uterus of mice in each group. Compared with the control group and control + catechins group, the expression of p-IRS1 and p-PI3K in the uterus of model group was significantly reduced. And catechins treatment increased the expression of p-IRS1 and p-PI3K in the uterus of model mice (Fig. 8c, e–f). These results indicated that catechins treatment may reduce PCOS symptoms and insulin resistance in model mice by inhibiting STAT3 signaling in the uterus of mice with PCOS.

## Discussion

Using the method of insulin resistance combined with hormonal disorders [29], we firstly replicated a PCOS mouse model [30], where we then showed that the health care effect of catechins extract on PCOS was related to the anti-inflammatory and anti-matrix degradation effects in the mouse uterus. Importantly, we demonstrated that the continuing healthcare benefit of catechins extract was to rescue hormonal disorders, insulin resistance, and ovarian and uterine pathological changes of PCOS mice by inhibiting STAT3 signaling in the uterus. We also found that catechins extract increased the IRS1 and PI3K signals in the uterus, inhibited the expression of MMP2 and MMP9, reduced the degradation of the endometrial matrix to change the uterine cell morphology, and down-regulated NF-κB to reduce uterine inflammation.

In addition to disorders of sex hormones, insulin resistance is also a common manifestation of PCOS, accounting for approximately 35% -40% of patients with PCOS [31]. Disorders of sex hormones and insulin resistance both play key roles in promoting the occurrence and progression of PCOS [16, 32]. Normal pulsatile secretion of LH is disrupted in patients with PCOS [33]. The proliferation of follicular membrane cells in patients with PCOS results in excessive androgen production and significantly enhances the stimulation of LH, leading to excessive secretion of LH [34]. Disorders of sex hormones and hyperinsulinemia are equally important in the development of PCOS [35]. They are positive feedback regulators of each other. Their complexity and extensiveness of organs are the difficulties in the clinical treatment of patients with PCOS [36]. Extensive studies have reported the healthcare effects of oolong tea extracts on obesity [37], but no researchers have focused on the healthcare effects of the extracts on PCOS. Consistent with our predictions, catechins extract not only significantly promotes glucose metabolism and insulin resistance without adverse effects on normal animals, but also had inhibitory effects on PCOS, including reducing the weight of uterus and ovary of PCOS mice, and regulating the sex hormone disorders in PCOS mice.

During the quality control of catechins in the experiment, we tested four substances and analyzed their contents, including Epigallocatechin (22.4 mg/g), Epicatechin (5.9 mg/g), Catechin (17.68 mg/g), and Epicatechin gallate (3.86 mg/g). Current research had shown that these substances have significant immune and anti-inflammatory effects [38], as well as their regulation of endocrine function. Recent research shows that the tea catechin epigallocatechin (gallate) can inhibit NF-κB-mediated transcriptional activation [39], another recent shows epicatechin gallate can protect HBMVECs from ischemia–reperfusion injury by promoting neurovascular regeneration [40]. The dietary intake of catechin and epicatechin can reduce vascular damage in high homocysteine intake mice, and reduce endothelial dysfunction and endothelial inflammation [41]. Therefore, we speculate that catechins has healthcare effect on the pathological changes of the ovaries during PCOS. As we have observed, catechins improve ovarian morphology in model mice. We also observed that the poor morphological changes of the uterus are also reversed by catechins in PCOS mice.

The process of implantation and pregnancy requires the coordinated interaction of [42]. Impaired endometrial function may cause recurrent miscarriages, preterm births, endometrial hyperplasia, and cancelation in patients with PCOS [43]. There is a correlation between inflammation-related molecular disorders and endometrial damage under PCOS [44]. We here focused on PCOS-related uterine dysfunction. After observing the endometrial damage of PCOS mice, we performed inflammatory factor-related tests on the uterus of mice. High levels of phosphorylated NF-κB in uterine tissues suggested an increase in uterine inflammation. Chronic low-grade inflammation plays an important role in the pathogenesis of PCOS [45]. Patients with PCOS clearly have increased serum C-reactive protein (CRP) and pro-inflammatory cytokine levels, including TNF-α, IL-1β, and IL-6 [46]. The uterus and ovary are physically linked biological tissues, and increased uterine inflammation has a pro-inflammatory effect on the ovaries. While observing that catechins can inhibit uterine inflammation in PCOS mice, we found that the expressions of MMP2 and MMP9 in serial sections of the uterus were also significantly reduced. Combined with the results of mouse uterine HE staining, these results suggest that the inhibitory effect of catechins on endometrial destruction is related to its ability to reduce MMPs.

STAT3 activation is caused by a variety of cytokines and growth factors, including activation by IL-6 signaling [47]. Studies have shown that activation of STAT3 in ovarian cancer leads to upregulation of MMP2 expression [48], and inhibition of STAT3 in pancreatic cancer cells results in down-regulation of MMP9 expression [49]. Some researchers reviewed STAT3 and NF-κB collaboration and crosstalk [50]. Both NF-κB and STAT3 can be rapidly activated in response to various stimuli (including IL-1β, IL-6, TNF-α). STAT3 may bind to NF-κB that bound IκB in an unphosphorylated form, replacing IκB from NF-κB, thereby contributing to NF-κB activation and nuclear entry even without conventional IKK signaling. We found that the expression of p-STAT3 in the uterus of PCOS mice was significantly increased, and the treatment of catechins reduced the level of p-STAT3 in the uterus of mice. The decrease of STAT3 signal may be the reason why catechins could inhibit the endometrial inflammation and reduce matrix degradation in PCOS mice.

PI3K is a lipid kinase that controls the core signal-regulating network in cells. PI3K/AKT signaling pathway is an important pathway in glucose metabolism. Insulin promotes glucose uptake through a series of signaling events triggered by binding to the insulin receptor and activating IRS1 phosphorylation, and IRS1-PI3K signaling is suppressed during insulin resistance. The proteomics data revealed the interdependence of PI3K and STAT3. PI3K and STAT3 are often accompanied by common activation in tumors, but STAT3 phosphorylation can also be independent of the PI3K pathway and induced by other mechanisms [51]. In this study, we observed that the STAT3 signal in the uterus of PCOS mice was opposite to the expression of p-IRS1 and p-PI3K, the expression of p-IRS1 and p-PI3K in the uterus of PCOS mice was significantly reduced, which was reversed by catechins.

## Conclusions

Our present study revealed that catechins reduced sex hormone disorders and insulin resistance in PCOS mice. Catechins down-regulate p-NF-κB p65-mediated inflammation in the uterus and inhibit MMP2 and MMP9-mediated endometrial damage, which improves uterine function in PCOS mice, and the inhibition of STAT3 signaling and up-regulation of IRS1/PI3K expression by catechins may play a key role on insulin resistance and uterine function in PCOS mice.

## Data Availability

The data used to support the findings of this study are available from the co-responding author upon request.

## Abbreviations

PCOS: Polycystic ovary syndrome
hCG: Human chorionic gonadotropin
OGTT: Oral glucose tolerance test
HOMA-IR: Homeostatic model assessment of insulin resistance
STAT3: Signal transducer and activator of transcription 3
NF-κB: Nuclear factor kappa-B
IRS1: Insulin receptor substrate 1
PI3K: Phosphoinositide 3-kinase
MMP: Matrix metallopeptidase
IKK: IκB Kinase
IL: Interleukin;
TNF-α: Tumor necrosis factor α
E2: Estradiol
FSH: Follicle-stimulating hormone
LH: Luteinizing hormone
